# Tailoring of Aqueous-Based Carbon Nanotube–Nanocellulose Films as Self-Standing Flexible Anodes for Lithium-Ion Storage

**DOI:** 10.3390/nano9040655

**Published:** 2019-04-24

**Authors:** Hoang Kha Nguyen, Jaehan Bae, Jaehyun Hur, Sang Joon Park, Min Sang Park, Il Tae Kim

**Affiliations:** 1 Department of Chemical and Biological Engineering, Gachon University, Seongnam-si, Gyeonggi-do 13120, Korea; hoangkhahc11dk@gmail.com (H.K.N.); jhbae1210@naver.com (J.B.); jhhur@gachon.ac.kr (J.H.); psj@gachon.ac.kr (S.J.P.); 2 B&I R&D Center, SK Innovation, 325, Exporo, Yuseong-gu, Daejeon 34124, Korea

**Keywords:** lithium-ion batteries, carbon nanotubes, nanocellulose, self-standing composite anode, carbonization

## Abstract

An easy and environmentally friendly method was developed for the preparation of a stabilized carbon nanotube–crystalline nanocellulose (CNT–CNC) dispersion and for its deposition to generate self-standing CNT–CNC composite films. The composite films were carbonized at different temperatures of 70 °C, 800 °C, and 1300 °C. Structural and morphological characteristics of the CNT–CNC films were investigated by X-ray diffraction (XRD), Raman spectroscopy, and scanning electron microscopy (SEM), which revealed that the sample annealed at 800 °C (CNT–CNC_800_) formed nano-tree networks of CNTs with a high surface area (1180 m^2^·g^−1^) and generated a conductive CNC matrix due to the effective carbonization. The carbonized composite films were applied as anodes for lithium-ion batteries, and the battery performance was evaluated in terms of initial voltage profile, cyclic voltammetry, capacity, cycling stability, and current rate efficiency. Among them, the CNT–CNC_800_ anode exhibited impressive electrochemical performance by showing a reversible capacity of 443 mAh·g^−1^ at a current density of 232 mA·g^−1^ after 120 cycles with the capacity retention of 89% and high rate capability.

## 1. Introduction

The bi-functionality of carbon nanotubes (CNTs), i.e., their anodic activity and their role as flexible supports to provide a conductive pathway, is the reason for their application in electrode materials [1,2,3], given the growing interest for high-capacity and flexible energy storage and especially for lithium-ion batteries (LIBs). Lithium ions are stored on the surface or in the interior of CNTs, and their large specific surface area is favorable since it provides more space for electrolyte ion adsorption [1]. When combined with ion storage capacity, a deposit of CNTs creates a porous interconnected network that facilitates ion and electron transport and provides mechanical durability [2,3], thereby achieving a highly capable current-free anode [4].

Despite its potential, the CNT-based anode is seldom practically used in the LIB industry. In LIB manufacturing, an electrode is fabricated by casting slurry in which active materials, binders, and conductive additives are mixed together [5]. The CNT exhibits strong van der Waals interactions between nanotube sidewalls, leading to aggregates [6]. Thus, the use of an organic solvent is required, and this leads to both safety concerns and increases in production cost, thereby impeding its commercial use in LIBs [7]. Although several routes toward CNT dispersion in water, including chemical functionalization [8] and addition of surfactant or polymer [9], were proposed, each method exhibits a corresponding disadvantage. For example, chemical functionalization disrupts the electronic structure of the pristine nanotubes and decreases conductivity [8], and the addition of inactive materials causes a surface coverage of the CNTs, weakening inter-tubular contact for electron hopping [9]. Therefore, a method of preparing a homogenous and uniform dispersion of CNTs in water via a facile, low-cost, and green approach still remains as a challenge.

Nanocellulose attracted interest in energy storage research and is employed as a constitutive component in LIB electrodes [10]. Characteristics of nanocellulose, including nanoscale dimension and reactive surface chemistry, are conducive for combining it with nano-sized active materials, thereby providing a high specific surface area [11]. Nanocellulose also exhibits an excellent colloidal stability in water [12] and enables carbon nanomaterials, such as CNT and graphene, to disperse in aqueous media without chemical functionalization or addition of surfactant/water-soluble polymers [9,13]. Utilizing these benefits, nanocellulose was applied as a binding constitute to construct pliable self-standing CNT electrodes via the aqueous solution process. However, most electrodes were prepared by mixing active materials with nanocellulose with fibril foam, such as cellulose nanofibril (CNF) or bacterial cellulose (BC), due to their entangled web-like structure [14,15,16], and this can lead to relatively high interfacial resistance between active materials which suffers from the low dispersion limit of CNTs in aqueous media.

In the present study, we focused on nanocellulose with whisker foam, namely crystal nanocellulose (CNC). We exploited the merits of CNC including (1) a higher specific surface area than CNF or BC [11], and (2) a high crystalline structure that facilitates CNC’s conversion into a conductive carbon by carbonization [17], which creates the binder-free effect. In the study, we developed an easy and environmentally friendly protocol involving the preparation of a stabilized CNT–CNC dispersion and its deposition to prepare a self-standing CNT–CNC film followed by carbonization. We examined the role of CNC in the composite film and its morphology changes during the carbonization. Carbonized composite films were applied as an anode and their battery performance was evaluated in terms of capacity, cycling stability, and current rate (C-rate) efficiency.

## 2. Materials and Methods 

### 2.1. Preparation of CNC, CNT-CNC, and CNT Electrodes

**Figure 1:**
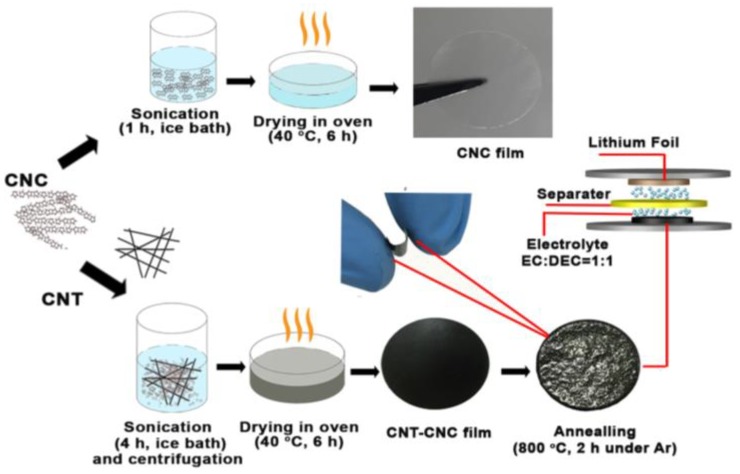
Schematic illustration of the free-standing electrode fabrication process.

CNCs (freeze-dried powders; Process Development Center, University of Maine, Orono, ME, USA) with an average length of 120 nm and aspect ratio of 10 were dispersed in deionized (DI) water (0.2 g CNC/100 g DI water), and the surface charge of CNC was dependent on pH (Figure S1, Supplementary Materials), which affects the dispersion and stabilization of CNT (OCSIAL LLC, Columbus, OH, USA) [13]. The CNT–CNC dispersion was prepared by mixing CNT (single-walled nanotubes) powders in the CNC–water mixture at pH 6.5 via a bath-type ultrasonication (Figure 1). 

Self-standing CNC films were simply prepared by drying the deposit dispersion consisting of 10 mL of solution in the petri dish at 40 °C for 6 h, and this was followed by vacuum-drying at 70 °C for 12 h to remove moisture (hereafter, the CNC film is denoted as CNT–CNC_70_). For the electrode annealed at 1300 °C, 40 mL of dispersion was used in order to obtain the well-maintained free-standing electrode, due to significant decomposition of materials at high temperature. The self-standing capability of CNT–CNC_70_ was evaluated as ductile and sufficiently robust to withstand bending or compression. For the heat treatment, CNC_70_ was annealed at 800 °C and 1300 °C [17]. In brief, the CNC_70_ electrode was heated to 240 °C with the heating rate of 5 °C∙min^−1^ and stabilized at 240 °C for 4 h in an Ar atmosphere. Afterward, it was heated to 800 °C (CNC_800_) or 1300 °C (CNC_1300_) with the rate of 5 °C·min^−1^ based on the extent of decomposition and graphitization of the composite films (discussed later), respectively, and maintained at the designated temperature for 2 h. A similar procedure was applied for CNT–CNC electrodes, accordingly denoted as CNT–CNC_70_, CNT–CNC_800_, and CNT–CNC_1300_. To compare the CNT–CNC electrodes, a bare CNT electrode was also formed by using pure CNT and a polyvinylidene fluoride (PVDF) binder. CNTs were mixed with PVDF at a weight ratio of 70:30, and *N*-methyl-2-pyrrolidone (NMP) was used as the solvent [18]. Specifically, CNT slurry was spread on a hydrophobic membrane and then compressed under a pressure of 10 atm·cm^−2^ for 30 min to form an electrode. The hydrophobic membrane was peeled after compression. Finally, the electrode was annealed at 70 °C, 800 °C, and 1300 °C. Each sample was denoted as CNT_70_, CNT_800_, and CNT_1300_, respectively. With respect to the electrochemical test, the CNT_800_ sample was used as a bare CNT. 

### 2.2. Material Characterization

We evaluated the quality of CNT dispersion by exploiting the visible absorption, and we determined the amount of CNT that was stably dispersed in the CNC–water mixture based on the Beer–Lambert law [19] (see details Supplementary Materials). The ratio of CNT:CNC was identified by thermogravimetric analysis (SDT Q600 V20.9 Build 20). Resistance and conductivity measurements of the CNT–CNC films were performed using the four-point probe technique (CMT-SR1000N). The structural characteristics of the as-prepared samples were investigated by X-ray diffraction (D/MAX-2200 Rigaku, Tokyo, Japan) in the range of 20–80° in the 2θ mode at the scan rate of 2°∙min^−1^. A micro Raman spectrometer (ANDOR Monora500i, 633 nm) was used to evaluate the degree of crystallinity of CNT–CNC films. Morphologies of the free-standing electrodes were examined using a scanning electron microscope (SEM S-4700, Hitachi, Tokyo, Japan). For characterizing the cross-sectional morphology of the samples, they were immersed in liquid nitrogen and cryo-fractured.

### 2.3. Electrochemical Analysis

In order to examine the electrochemical properties of composite films, free-standing films at various conditions were utilized as potential anodes. After the process, each CR2032-type coin cell was assembled in an Ar-filled glove box with Li as the counter electrode, a sample electrode as the working electrode, and a polyethylene separator. Galvanostatic charge/discharge electrochemical tests were conducted at a constant current density of 232 mA·g^−1^ at 0.01–2.0 V vs. Li/Li^+^ via a battery cycler (WBCS3000, WonAtech). The rate cyclic performances were tested at different charge and current densities corresponding to 100 mA·g^−1^, 500 mA·g^−1^, 1000 mA·g^−1^, and 5000 mA·g^−1^ under a constant discharge current density of 100 mA·g^−1^ in order to analyze fast-charge characteristics. Cyclic voltammetry testing was performed via a ZIVE MP1 (WonAtech) device in the range of 0.01–2.0 V vs. Li/Li^+^ at a scanning rate of 0.1 mV·s^−1^. Electrochemical impedance spectroscopic (EIS) analysis was conducted via the ZIVE MP1 (WonAtech) analyzer by applying a 1-mV-amplitude signal in the frequency range of 100 kHz to 100 MHz. The impedance response was measured after varying the number of cycles at 2.0 V vs. Li/Li^+^. 

## 3. Results and Discussion

The prepared CNT–CNC dispersion shown in Figure S2 (Supplementary Materials) was stable even after a month of aging, while CNTs precipitated within an hour when similarly dispersed in pure water without CNCs; thus, it was unable to fabricate a bind-free anode. The determined weight ratio of CNT to CNC in the CNT–CNC dispersion was 100:11.21, as shown in Figure S3 (Supplementary Materials). The stable dispersion was attributed to the interaction between CNC and CNT as reported by Wagberg et al. [13] who hypothesized that counter-ion fluctuations on the nanocellulose surface cause assembly with CNTs and stabilize CNTs in aqueous media. Conversely, the CNT film prepared using the aqueous CNT suspension was fragile and broke easily when bent or compressed, which limited its use in the flexible electrode. The lack of flexibility was attributed to the disentanglement of CNTs. The result implied that well-dispersed CNTs in the CNC–water mixture facilitated stable networks during water evaporation. 

**Figure 2:**
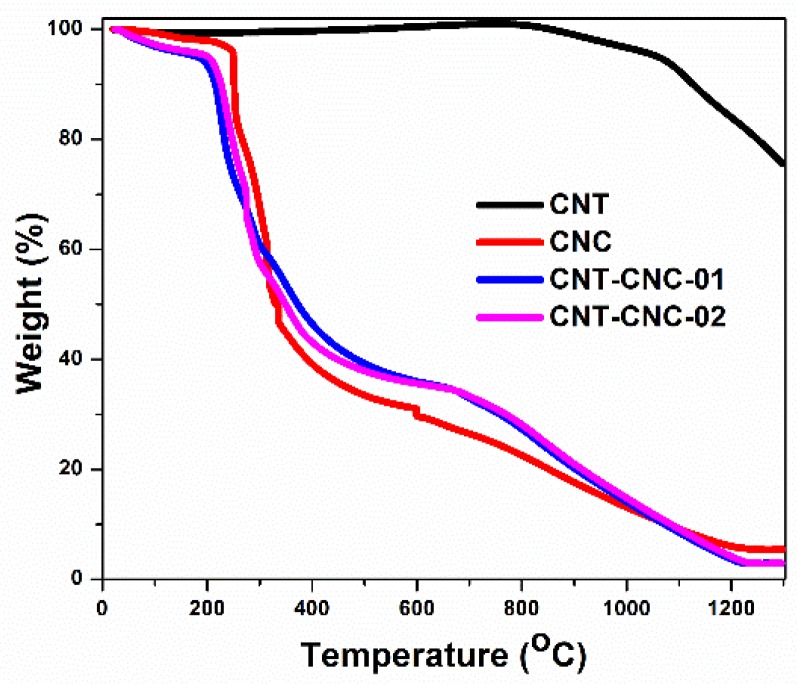
Thermogravimetric analysis data of carbon nanotube (CNT), crystalline nanocellulose (CNC), and CNT–CNC.

The pyrolysis of CNC was conducted to convert the insulating CNC into conductive nanocarbons. Specifically, thermogravimetric analysis (TGA) was performed under inert atmosphere (Ar) to investigate the weight loss of both precursors, namely CNC and CNT, during pyrolysis. CNT–CNC-01 and CNT–CNC-02 represent the as-prepared CNT–CNC composites with weight ratio of 100:11.21 for CNT and CNC in Figure 2, which were used for calculating the composition of CNT and CNC as shown in Tables S1, S2, and S3 (Supplementary Materials). The CNC exhibited the main weight loss in the temperature range of 250–400 °C, at which depolymerization of cellulose occurs along with dehydration [20]; thereafter, the weight loss slowly progressed (Figure 2). The results indicated that the weight loss of CNT began at approximately 800 °C and exhibited an increased weight loss with increases in temperature. Based on the result, we selected two points of temperature for pyrolysis, namely 800 °C at which CNC involves a change in chemical composition while CNT is not significantly affected, and 1300 °C as a designated temperature at which both CNC and CNT undergo thermal decomposition.

**Figure 3:**
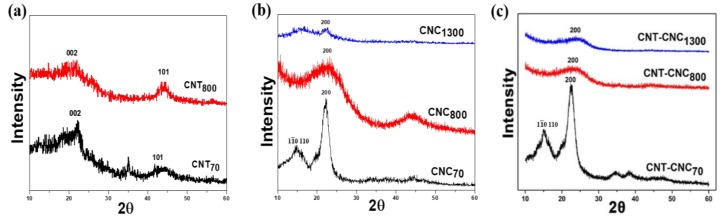
X-ray diffraction (XRD) pattern of (**a**) CNT at 70 °C and 800 °C, (**b**) CNC at 70 °C, 800 °C, and 1300 °C, and (**c**) CNT–CNCs at 70 °C, 800 °C, and 1300 °C.

We evaluated the effect of temperature on the structural change in the precursors, as shown in Figure 3. In the case of CNT composite films, the XRD patterns were not significantly different (Figure 3a). When looking into the peak at ~44°, it showed a very broad peak and a maximum peak intensity at ~44°, which was related to the (101) plane of CNT. In addition, the peak at ~25° corresponding to the (002) plane was also detected. It was noted that, when CNT was heat-treated at 800 °C, the peaks related to the CNT phase did not show significant changes. The comparison of XRD patterns of the CNC films indicated a broad peak at ~15°, related to and (110) planes [21], while a difference in the peak at *2θ* = 22.2° was assigned to the (200) plane. The CNC showed a broad peak at *2θ* = 22.2° after heat treatment at 800 °C, thereby indicating a very low crystallinity. Meanwhile, when comparing the XRD peak of CNC_1300_ at ~22° to that of CNC_800_, the CNC_800_ sample showed a very broad peak over the range of ~10° due to the very low crystallinity. On the other hand, the CNC_1300_ sample exhibited an abrupt small peak at ~22°, which could be due to a partial graphitization in a small portion of CNC. This explanation is in good agreement with the Raman result, where the I_G_/I_D_ value of CNC_1300_ (1.36) was higher than that of CNC_800_ (0.99) (discussed later). This implied that crystallized cellulose, i.e., CNC, was carbonized at 800 °C and tended to form graphitized carbon during carbonization at 1300 °C. The partial graphitization that was achieved at the relatively low temperature of 1300 °C was related to the highly crystalline structure of CNC. For the CNT–CNC composite films, the XRD pattern of CNT–CNC_70_ was similar to that of CNC_70_. It was noted that the peaks related to CNT, as shown in Figure 3a, were not detected in CNT–CNC composite films (Figure 3c), which could be due to the very small amount of CNT in CNC. After being heat-treated at 800 °C and 1300 °C, the peaks related to CNC functional groups disappeared and a broad peak corresponding to carbonized carbon peaks was observed. 

**Figure 4:**
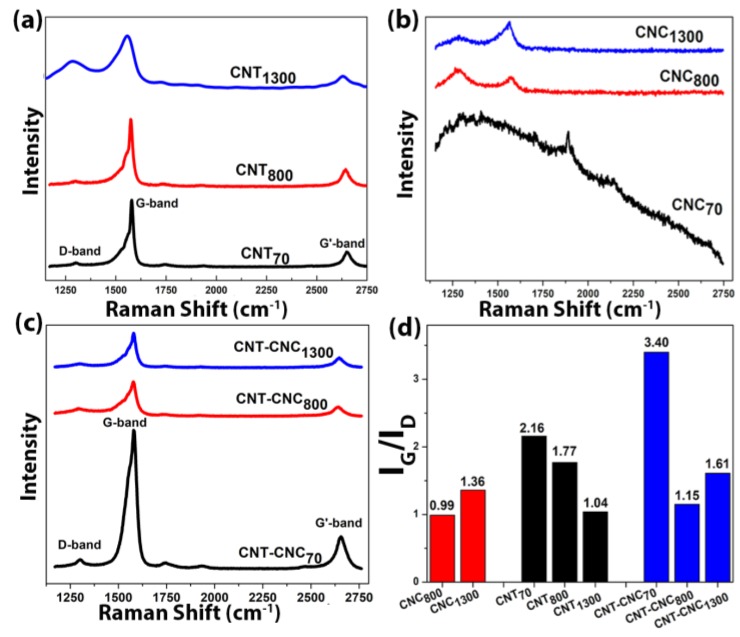
Raman spectra of (**a**) CNT at 70 °C, 800 °C, and 1300 °C, (**b**) CNC at 70 °C, 800 °C, and 1300 °C, and (**c**) CNT–CNCs at 70 °C, 800 °C, and 1300 °C. (**d**) Comparison of I_G_/I_D_ ratio with various samples.

Carbonization of CNC was also confirmed by the appearance of Raman bands that characterize *sp*^2^ hybridized carbon, i.e., the G-band (approximately 1580 cm^−1^) and D-band (approximately 1350 cm^−1^) after pyrolysis (Figure 4a–c). The intensity ratio of G-band and D-band CNT, I_G_/I_D_, was approximately 2.0 before hydrolysis and slightly decreased to 1.8 and to 1.0 with heat treatments at 800 °C and at 1300 °C (Figure 4d), respectively. The result implied a higher portion of defects generated via heat treatment (discussed later). 

The results of the controlled experiments indicated that the composition ratio of CNT to CNC in CNT–CNC_800_ and CNT–CNC_1300_ was 0.57:0.43 and 0.24:0.76, respectively (the weight loss of CNC and CNT based on the temperature is described in Tables S1–S3, Supplementary Materials). The resultant CNT–CNC films did not shrink in plane after the pyrolysis, and the thickness of the composite changed from 70 μm (CNT–CNC_70_) to 4 μm (CNT–CNC_800_) and 13 μm (CNT–CNC_1300_). It was noted that the CNT–CNC_1300_ electrode was thicker than the CNT–CNC_800_ electrode after pyrolysis, which could be due to the higher amount of composite solution applied. Practically, when a low amount of solution was used for the CNT–CNC_1300_ electrode, it was hard to develop good free-standing electrodes. The resultant films retained their flexibility (Figures S4–S5, Supplementary Materials). The result implied that the thermal decomposition did not significantly damage the inter-connected network of CNTs in plane, and that the volume change caused by CNC decomposition was offset by the compression along the thickness.

**Figure 5:**
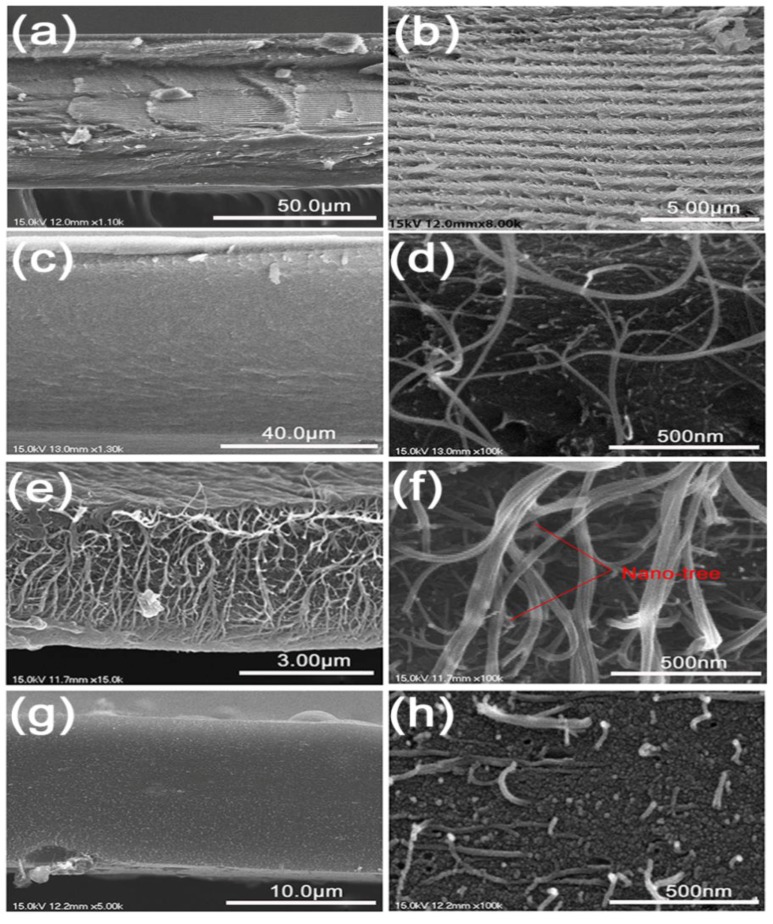
Cross-section morphologies of (**a**,**b**) CNC_70_, (**c**,**d**) CNT–CNC_70_, (**e**,**f**) CNT–CNC_800_, and (**g**,**h**) CNT–CNC_1300_.

Pyrolysis changed the morphology of the composite films based on the temperature. A comparison of the cross-section SEM images of bulk-CNC, CNT-CNC_70_, CNT-CNC_800_, and CNT-CNC_1300_ indicated the clear difference in morphology (Figure 5). 

The pure CNC film presented a chiral nematic nanostructure that was preserved from a mesophase of CNC suspension [22,23] on slow evaporation (Figure 5a–b). However, with respect to CNT–CNC_70_, CNCs did not exhibit chirality and, instead, formed a dense matrix embedding CNTs (Figure 5c–d). When looking into the Brunauer–Emmett–Teller (BET) results (Figure S8, Supplementary Materials), the CNT–CNC_800_ sample demonstrated the highest N_2_-sorption isotherm with a high pore volume (Table S4, Supplementary Materials) and various pore diameters compared to other samples. It was noted that surface area of the CNT–CNC_1300_ sample was much lower than that of the CNT–CNC_800_ sample, where the calculated surface area corresponded to 1184.2 m^2^·g^−1^ for CNT–CNC_800_ and 278.2 m^2^·g^−1^ for CNT–CNC_1300_. The different form of the isotherms and the significant change of the surface area between CNT–CNC_800_ and CNT–CNC_1300_ samples could be due to the distinguishable morphologies. In the morphology of CNT–CNC_800_, large empty spaces were formed between the inter-connected three-dimensional (3D) networks of intact CNTs (Figure 5e–f). Under the static in-plane dimension by the CNT network, the loss of CNC appeared to generate the spaces. However, the cross-section of CNT–CNC_1300_ exhibited a different morphology, wherein short CNTs with a broken end were embedded in the coarse CNC matrix (Figure 5g–h) (see Figures S6 and S7, Supplementary Materials, for further morphology data). This explanation is in good agreement with the BET results showing a high surface area in the CNT–CNC_800_ composite film.

**Figure 6:**
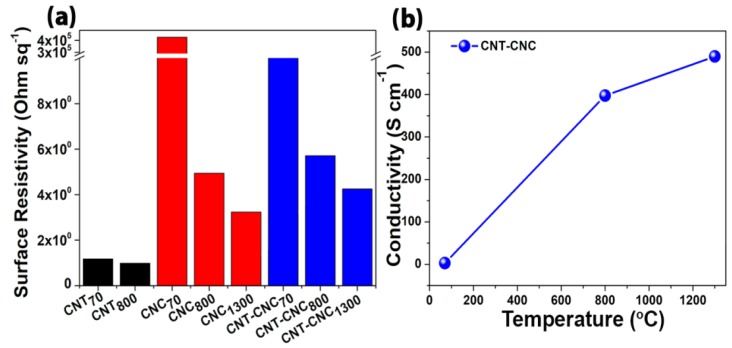
(**a**) Surface resistivity of various samples. (**b**) Conductivity of CNT–CNCs with different annealing temperatures.

Electrical conductivity was also significantly affected by the heat treatment. CNT–CNC_70_ exhibited almost non-conductive behavior (electric conductivity of approximately 0 S·cm^−1^) (Figure 6), presumably because several insulating CNCs adhered to the surface of the CNTs and, thus, did not reach critical percolation. Conversely, as shown in the morphology of CNT–CNC_800_, the restored long-range connectivity of CNTs recreated an electron-conducting pathway, thereby enhancing electrical conductivity (approximately 400 S·cm^–1^). The higher conductivity of CNT–CNC_1300_ (approximately 500 S·cm^−1^) was attributed to the fact that the partially graphitized carbonaceous CNC (which is conducive and able to closely contact with CNTs due to their strong π–π interactions [24]) was “welded” into the surface of CNTs, thereby significantly lowering the contact resistance despite the existence of broken CNTs.

The as-prepared materials were applied as anodes for lithium-ion batteries. The charge/discharge voltage profiles of as-prepared electrodes were analyzed. Firstly, we evaluated anodic activity using pure CNC and CNT. It was noted that electrochemical reactions of the CNC_70_ electrode were absent, and this illustrated the absence of electrochemically active components. In the case of the CNC_800_ electrode, the first discharge and charge capacities were 168 mAh·g^−1^ and 130 mAh·g^−1^, respectively, corresponding to a Coulombic efficiency of 77%, while the CNC_1300_ electrode exhibited a discharge/charge capacity of 236 mAh·g^−1^/130 mAh·g^−1^ with a Coulombic efficiency of 55% (Figures S9b–c, Supplementary Materials). Additionally, CNT electrodes exhibited a higher Coulombic efficiency of 91%. However, low initial discharge and charge capacities of 250 mAh·g^−1^ and 227 mAh·g^−1^, respectively, were obtained (Figure S9a, Supplementary Materials). 

**Figure 7:**
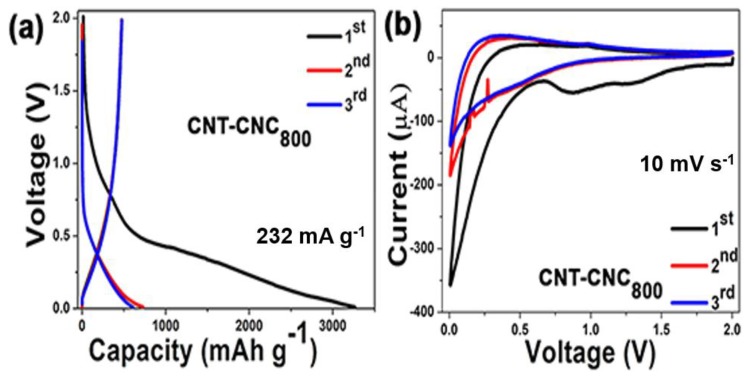
Electrochemical performance data of the composite electrodes. (**a**) Initial voltage profiles of the CNT–CNC_800_ electrode. (**b**) Cyclic voltammograms of the CNT–CNC_800_ electrode.

Conversely, with respect to the CNT–CNC composite electrodes, Figure 7a shows the charge/discharge initial voltage profiles of CNT–CNC_800_ at a current density of 232 mA·g^−1^ with a voltage range from 0.01 to 2.0 V. The initial discharge and charge capacities were observed as 3280 and 478 mAh·g^−1^, respectively, corresponding to a low initial Coulombic efficiency of 14.6%. This irreversible capacity of the CNT-based electrodes could be limited by the control of the inserted lithium [25]. The discharge/charge capacities quickly decreased during the next few cycles and maintained a reversible capacity of 608 mAh·g^−1^ to form the third cycle. The phenomenon was attributed to various reasons such as the formation of the solid electrolyte interphase (SEI) layer on the surface of the electrode [26,27,28], the oxygenated functional group on the surface electrode [28], and intercalation of Li into the inner core and side wall of CNT [29]. Based on the aforementioned voltage profile analysis, it mainly focused on the discussion of the composite electrode CNT–CNC_800_ with respect to the electrochemical performance. Figure 7b shows the initial cyclic voltammograms of the CNT–CNC_800_ electrode. The capacity–voltage (CV) curves of the CNT–CNC electrode indicated that lithium ions reversibly (de)intercalated into CNTs [29,30,31]. The CNT–CNC_800_ electrode exhibited a broad peak at approximately 0.1 V and 0.4 V in the initial cycle, and this can be related to the lithium deintercalated from the CNTs. Specifically, at the potential of 0.16 V, the current reached the highest point, thereby indicating the deintercalation of lithium ions from the carbon nanotube. Additionally, the peaks at 0.8 V and 1.25 V appeared in the first cycle and disappeared in the second cycle, thereby indicating the formation of the SEI layer. During the negative scan, the potential of lithium intercalated to CNTs approximately corresponded to 0.05 V, and this was extremely close to 0 V versus the Li^+^/Li reference. From the second cycle, the intensity of redox peaks was maintained, thereby illustrating stable and reversible electrochemical reactions.

**Figure 8:**
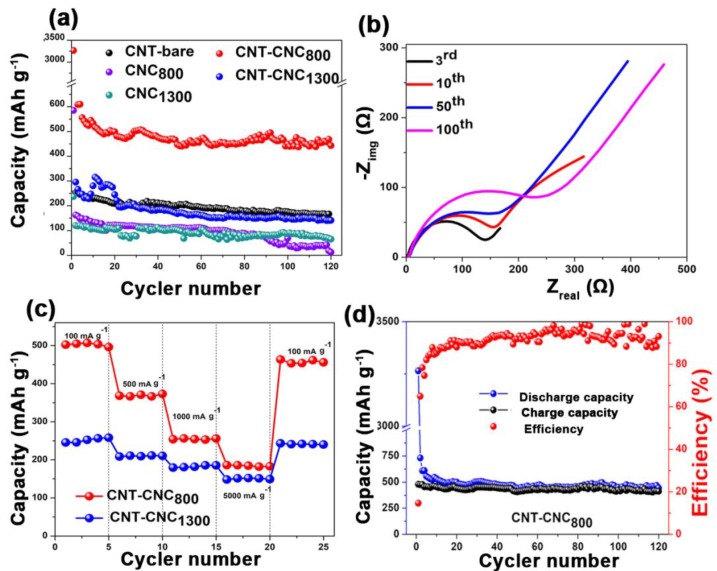
(**a**) Cyclic performance of bare CNT bare, CNC_800_, CNC_1300_, CNT–CNC_800_, and CNT–CNC_1300_. (**b**) Electrochemical impedance spectroscopic measurement for CNT–CNC_800_ after the third, 10th, 50th, and 100th cycles. (**c**) Rate performance of CNT–CNC_800_ and CNT–CNC_1300_. (**d**) Cyclic performance with respect to the Coulombic efficiency of CNT–CNC_800_ for 120 cycles.

Figure 8a shows the discharge capacities of various samples that were developed at different annealing temperatures including 70 °C, 800 °C, and 1300 °C. A free-standing bare CNT electrode developed with PVDF binder (CNT_800_) was also tested to compare the effectiveness of CNCs on the electrochemical performance. With respect to bare CNT, the capacity values were approximately 200 mAh·g^−1^. However, the capacity was low when compared to that of CNT–CNC_800_ (discussed below) due to the introduction of PVDF, which can act as an insulator although it can generate flexibility in the CNT film [32,33,34,35,36]. In the case of the sample with the annealing condition at 70 °C, it did not demonstrate any electrochemical reactions of the CNT–CNC electrode, and this resulted in a capacity corresponding to zero value. As shown in Figure 6, the result of conductivity confirmed that CNC at the annealing temperature of 70 °C exhibited extremely high resistance, and this was inappropriate for use as an electrode. It was noted that the electrochemical performance significantly improved with increases in the annealing temperature. For example, the CNC_800_ electrode exhibited an initial discharge capacity of 586 mAh·g^−1^. However, it exhibited a gradual decrease in capacity after a few cycles and decreased to zero at the 120th cycle. While conducting heat treatment at 1300 °C, there was also no significant improvement in capacities. Comparatively, in the case of CNT–CNC electrodes, the electrochemical performance was significantly different based on the applied annealing temperature. With respect to the CNT–CNC_1300_ electrode, the capacity was significantly lower than that of the CNT–CNC_800_ electrode, and this delivered a discharge capacity of 140 mAh·g^−1^ after 120 cycles. The capacity values of CNT–CNC_1300_ were similar to those of the bare CNT electrode. Given the application of extremely high temperature (i.e., 1300 °C), the CNC decomposed. In actuality, nearly 98% of CNC was degraded, as shown in Table S3 (Supplementary Materials). Furthermore, with respect to the SEM images in Figures S6h and S7h (Supplementary Materials) for the CNT–CNC_1300_ electrode, the CNT–CNT network was clearly reduced and broken. Furthermore, the electrode was extremely brittle and weakened. Meanwhile, with respect to the CNT–CNC_800_ electrode, it exhibited the highest capacity with significant stability, as shown in Figure 8a,d. Its discharge capacity was approximately 495 mAh·g^−1^, and it maintained a capacity of 443 mAh·g^−1^ after 120 cycles, thereby illustrating a good capacity retention of 89%. The SEM images of the CNT–CNC_800_ sample (Figure 5e,f) exhibited an interesting network morphology between CNC and CNT. The cross-section images proved that CNT formed a good network with the product of CNC decomposition during heat treatment. Additionally, it should be noted that CNTs exhibited a “nano-tree-like” shape with a good inter-connection and suitable distances between the trees (Figure 5f) [37]. Its morphology generated an increase in the surface area of the electrode, and this was approximately 1180 m^2^·g^−1^. Furthermore, 43% of the CNC was decomposed and disappeared based on the TGA results (Figure 2); thus, there were free spaces in the network. Therefore, the developed film was more anodic-capable, albeit still flexible. Furthermore, in the case of CNT–CNC_800_, CNT maintained its original structure well, which could have the ability to absorb two lithium layers per one carbon layer to form Li_2_C_6_ [38]. This phenomenon was comprehensively proven in a previous study. Numerous CNT edges absorb Li atoms; as a result, the theoretical capacity of the CNT–CNC electrode could exceed the theoretical capacity (372 mAh·g^−1^) and reach high capacity values (~744 mAh·g^−1^) [39,40,41]. Based on the information, it is proposed that the CNT–CNC electrode that is appropriately heat-treated exhibits high potential for the application of flexible LIBs.

Figure 8b shows the electrochemical impedance spectroscopic (EIS) images for the as-prepared electrode. The EIS analysis followed the modeling of R_s_-(R_ct_|W-Q_dl_) and Nyquist plot for CNT–CNC_800_ electrode after the third, 10th, 50th, and 100th cycles. The first semicircle at high frequency corresponded to the migration of Li ions via the passivation film (such as the SEI), and the second semicircle at the intermediate frequency corresponded to the charge transfer reaction [42,43]. The linear portion was attributed to semi-infinite diffusion conditions for the diffusion of lithium ions in carbon nanotubes [44] because the diffusion of lithium ions in the carbon nanotubes was considerably slower than that in an electrolyte solution [43]. It is noted that heat treatment significantly affected the resistance of the electrode for the CNT–CNC composite. For example, the R_ct_ value of CNT–CNC_70_ was extremely high (4710 Ω), and this was related to the absence of electrochemical reactions as discussed earlier. Additionally, the CNT–CNC_1300_ electrode indicated a mild increase in R_ct_ value corresponding to 241 Ω due to the effects of the CNT decomposition, while CNT–CNC_800_ exhibited an R_ct_ value of 134 Ω. The resistance values of CNT–CNC_800_ and CNT–CNC_1300_ were comparable to that of the CNT electrode (105 Ω), thereby illustrating the positive impact of heat treatment on decreasing the resistivity of the composite electrodes.

Furthermore, CNT–CNC electrodes exhibited excellent rate performances at different current densities ranging from 100 to 5000 mA·g^−1^, as shown in Figure 8c. The CNT–CNC_1300_ electrode showed an average capacity of 250 mAh·g^−1^ at a current density of 100 mA·g^-1^. When current density increased to 5000 mA·g^−1^, it maintained an average capacity of 150 mAh·g^−1^ corresponding to a capacity retention of ~60%. Meanwhile, in the case of the CNT–CNC_800_ electrode, the capacity at 100 mA·g^−1^ was approximately 500 mAh·g^−1^ with good stability. When the current density increased to 5000 mA·g^−1^, a reversible capacity of 182 mAh·g^−1^ was obtained by demonstrating a capacity retention of approximately ~40%. When the current density recovered to 100 mA·g^−1^, the CNT–CNC_800_ electrode still exhibited a good reversible capacity of 450 mAh·g^-1^ by demonstrating a capacity restoration of 90%. However, it was noted that, even though the capacity values of the CNT–CNC_800_ electrode at different current densities were higher than those of the CNT–CNC_1300_ electrode, the capacity retentions of the CNT–CNC_1300_ electrode based on the capacity values at 100 mA·g^−1^ were higher than those of CNT–CNC_800_. Therefore, even though both CNT–CNC_800_ and CNT–CNC_1300_ electrodes demonstrated good rate performance, we can draw a conclusion that the CNT–CNC_1300_ electrode showed better rate performance compared to the CNT–CNC_800_ electrode in terms of capacity retention, which could be due to the higher electrical conductivity of CNT–CNC_1300_ (Figure 6). Finally, we compared the performance of the CNT–CNC_800_ electrode with various flexible electrodes with CNTs, as shown in Table S5 (Supplementary Materials), and they exhibited simple preparation and superior electrochemical performance.

## 4. Conclusions

In the study, pliable self-standing CNT–CNC electrodes with significant electrochemical performance were achieved via a simple aqueous-based preparation method. Specifically, CNC was applied to act as the binding constitute to generate a self-standing CNT electrode, leading to stable dispersity of CNT in the CNC solution, and the heat treatment process introduced new characteristics, including conductive CNC and free spaces in the CNT–CNC structure. The sizes and structures of the pores and the porosity critically affected their battery performance. With respect to the electrodes, hierarchical microporous–mesoporous structures were desirable in terms of improving the contact area of the electrode–electrolyte, decreasing the diffusion resistance of electrolyte ions, and shortening the diffusion length of the electrolyte ions. Given this viewpoint, the porous structure of CNT–CNC_800_ exhibited outstanding electrochemical performance. However, it was recognized that carbonization and/or graphitization of CNT–CNC composite films at various annealing temperature are also important to achieve more optimized electrochemical performance, which could be the basis of future work. In summary, the CNT–CNC_800_ electrode developed in this study is a promising candidate for effective lithium-ion storage.

## Supplementary Materials

The following are available online at https://www.mdpi.com/2079-4991/9/4/655/s1: Figure S1: The dependence of surface charge on the pH; Figure S2: Illustration of (a) solution CNCs, (b) solution CNT–CNC, and (c) aqueous CNT; Figure S3: Ultraviolet (UV)–visible absorption spectra; Table S1: The initial composition of the electrode; Table S2: The composition of the electrode at 800 °C; Table S3: The composition of the electrode at 1300 °C; Figure S4: Images of free-standing electrodes with different annealing temperatures; Figure S5: Images of pliable electrodes; Figure S6: SEM images (surface section); Figure S7: SEM images (cross-section); Figure S8: Brunauer–Emmett–Teller (BET); Figure S9: Initial voltage profiles; Figure S10: Cyclic voltammograms; Figure S11: TEM images of CNTs; Table S4: Specific surface area of the as-prepared electrode films; Table S5: Comparison of the performance for various flexible electrodes with CNTs.
