# Supplementary material for: Tailoring of Aqueous-Based Carbon Nanotube–Nanocellulose Films as Self-Standing Flexible Anodes for Lithium-Ion Storage
Source: Nanomaterials (Basel). 2019 Apr 24;9(4):655. doi: 10.3390/nano9040655 (PMC6523255; doi:10.3390/nano9040655)
Supplement: Supplementary file 1 [file nanomaterials-09-00655-s001.pdf]

Supplementary information

Tailoring of aqueous-based carbon nanotubes-nanocellulose films as self-standing flexible anodes for lithium-ion storage

Hoang Kha Nguyen[1], Jaehan Bae[1], Jaehyun Hur[1], Sang Joon Park[1], Min Sang Park[2,**], Il Tae Kim[1,*]
[1] Department of Chemical and Biological Engineering, Gachon University, Seongnam-si, Gyeonggi-do 13120, Republic of Korea
[2] B&I R&D Center, SK Innovation, 325, Exporo, Yuseong-gu, Daejeon 34124, Republic of Korea
*Corresponding author. Tel.: +82-31-750-8835; fax: +82-31-750-5363
**Corresponding author. Tel.: +82-42-609-8910; fax: +82-02-2121-4001
E-mail addresses: azopark@gmail.com (M. S. Park) and itkim@gachon.ac.kr (I.T. Kim)

The dependence of surface charge on the pH

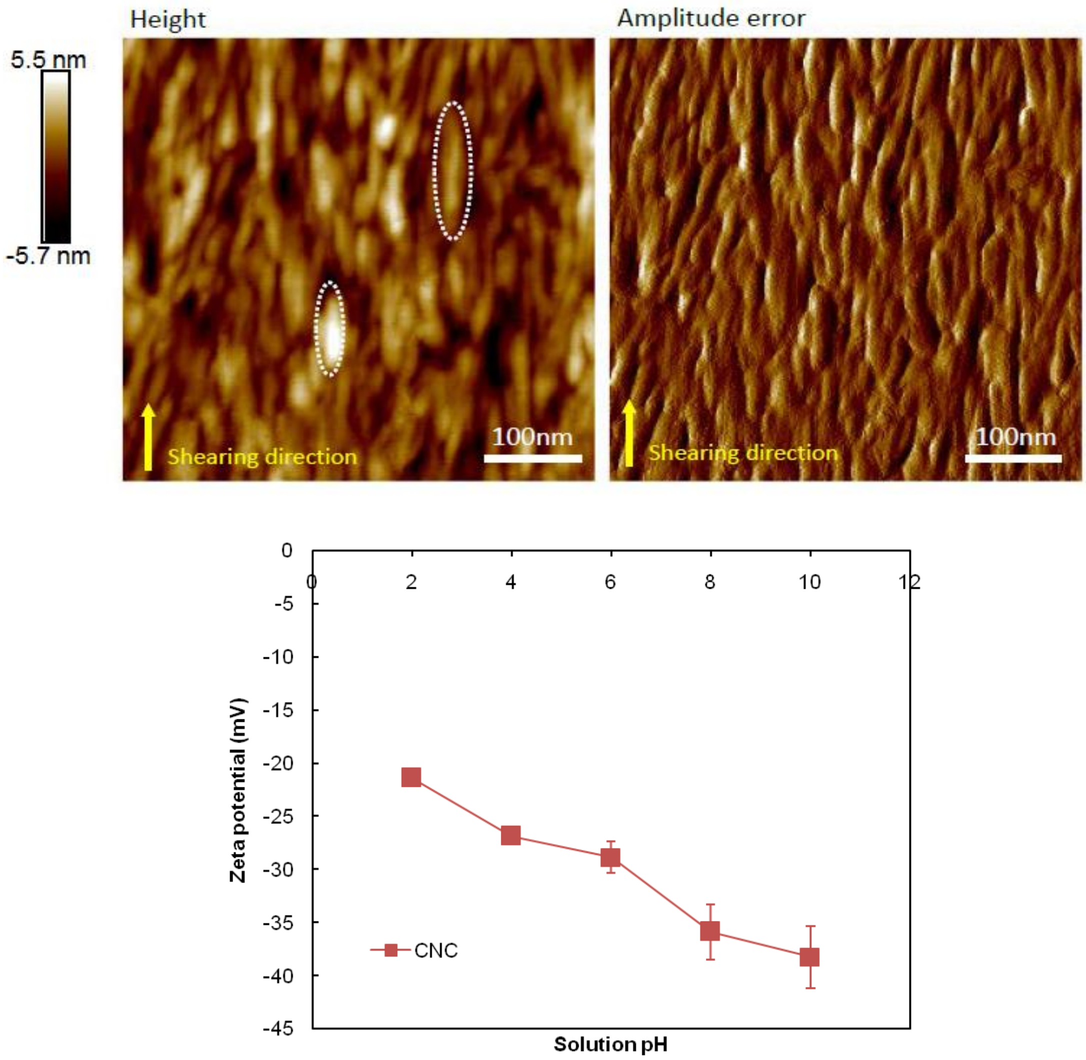

Figure. S1. The dependence of surface charge on the pH.

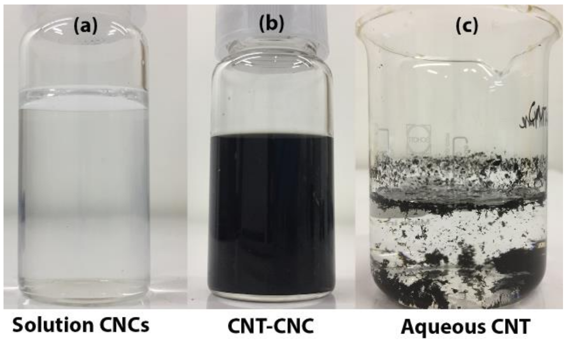

Figure. S2. Illustration of (a) Solution CNCs, (b) Solution CNT-CNC and (c) Aqueous CNT.

1. **Measurement of ultraviolet-visible spectroscopy**

Beer-Lambert's law was used to identify the concentration of the CNT-CNC solution. This is expressed as follows:

$$A = \varepsilon * b * c$$

where ε denotes the wavelength-dependent molar absorptivity, coefficient b denotes the path length, and c denotes the concentration of the solution.

After dispersing and sonicating CNT-COOH in 1000-ml water, the solution was filtered. Subsequently, dispersed CNT-COOH (C_o) penetrated the filter, and its weight was measured via the dry weight method as C_o= 8.8 ppm [1,2]. Eight samples in different concentrations were prepared to form the calibration line (at concentrations of $C_o/8$, $2C_o/8$, $3C_o/8$, $4C_o/8$, $5C_o/8$, $6C_o/8$, $7C_o/8$, and $8C_o/8$) as shown in Figure. S2(a). The calibration line is described by the equation: y = 0.0006x + 0.0259 (R^2 = 0.9979: acceptable). With respect to the measurement of CNT-CNC solution, the solution was diluted to a concentration of 0.1 g/l of CNC (10 ml of solution (CNT-CNC) in DI water) to measure the absorbance from UV-Vis spectroscopy. Based on the calibration line, $CNC/CNT_{solution}$ = 100 ppm: 11.21 ppm. With respect to obtaining the concentration of CNT-CNC, a wavelength corresponding to 500 nm was selected to maximize the transmittance of cellulose crystal and absorbance of carbon nanotubes [1-6].

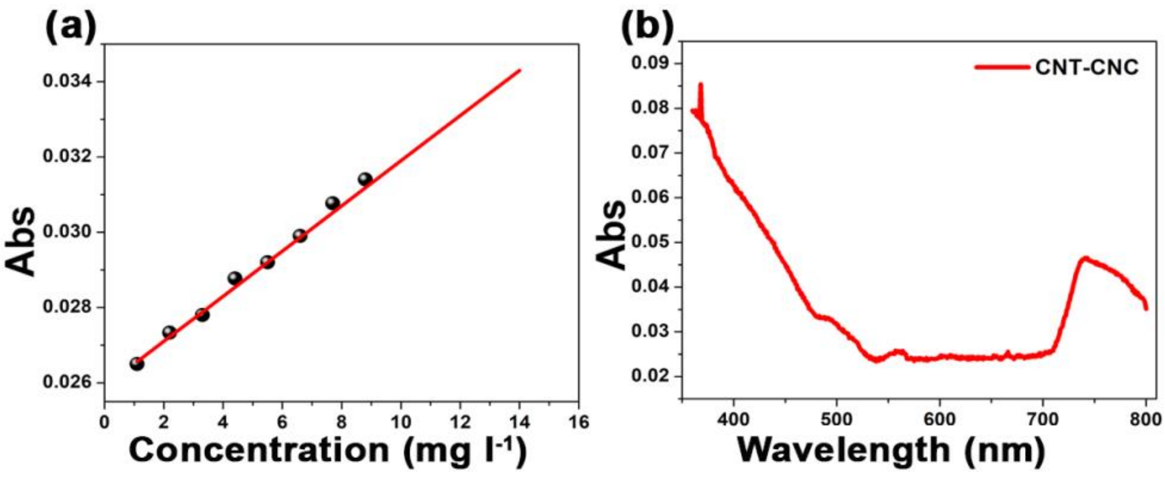

Figure. S3. UV-visible absorption spectra. (a) Calibration line of the CNT-COOH solution and (b) absorbance spectrum of CNT-CNC.

2. Thermal gravimetric analysis

The ratio of CNC and CNT in the composite films annealed at different temperatures was estimated via TGA analysis (SDT Q600 V20.9 Build 20). The decomposition of CNT began at approximately 800 °C while CNC and CNT-CNC film underwent decomposition at approximately 230 °C (Figure. 2).

With respect to the initial samples, the compositions of the electrodes are given in Table S1. It should be noted that the ratio of CNC: CNT = 100:11.21 as indicated by the UV-Vis experiment.

Table S1. The initial composition of the electrode.

	Initial mass of film (mg)	Moisture content (mg)	Initial mass of CNT-CNC (mg)	Initial mass of CNT (mCNT) (mg)	Initial mass of CNC (mCNC) (mg)
Sample 01	10.546	0.4107	10.1353	1.0185	9.1167
Sample 02	10.992	0.4466	10.5454	1.0598	9.4857

Table S2. The composition of the electrode at 800 °C.

	Total weight (mg)	Weight of CNT (mg)	Weight of CNC (mg)	wt% of CNT	wt% of CNC
Sample 01	2.3781	1.0186	1.3595	42.8	57.2
Sample 02	2.5102	1.0598	1.4504	42.2	57.8

Table S3. The composition of the electrode at 1300 °C.

	Total weight (mg)	Initial mass of CNT (mCNT) (mg)	Initial mass of CNC (mCNC) (mg)	K_1	K_2	wt% of CNT	wt% of CNC
Sample 01	0.5295	1.0186	9.1167	0.985	0.614	76.46	23.54
Sample 02	0.5509	1.0598	9.4856				

From the TGA data, it was assumed that the CNT was maintained without any degradation at 800 °C [4]. Based on the total weight and data from UV-Vis, the wt% of CNT and CNC is calculated as shown

in Table S2.

While observing the TGA graph, the decomposition of CNT began from approximately 800 °C. At 1300 °C, both CNC and CNT were decomposed. With respect to the calculation of the wt% of CNT and CNC, we applied the following equation (law of conservation of mass):

$$m_{CNC} \times (1-K_1) + m_{CNT} \times (1-K_2) = m_{material\ at\ 1300^oC} \quad (1)$$

where

m_{CNC} : weight of CNC in the initial film

m_{CNT} : weight of CNT in the initial film

$m_{material\ at\ 1300^oC}$: weight of material at 1300 °C

where K_1 and K_2 denote the proportions of mass loss of CNC and CNT, respectively. The weight composition of CNC and CNT at 1300 °C is obtained based on equation (1) where the calculated values are shown in Table S3. Thus, based on the TGA analysis, the weight percent of CNT : CNC is 10: 90 for $CNT\text{-}CNC_{70}$, 43: 57 for $CNT\text{-}CNC_{800}$, and 76:24 for $CNT\text{-}CNC_{1300}$.

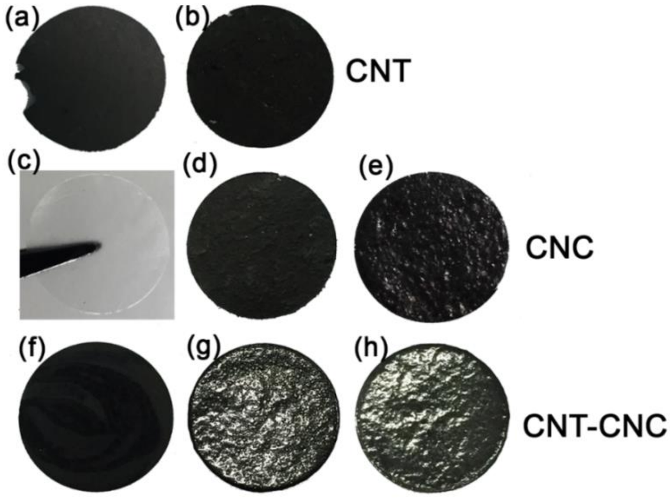

Figure. S4. Images of free-standing electrodes with different annealing temperatures: (a) CNT_{70}, (b) CNT_{800}, (c) CNC_{70}, (d) CNC_{800}, (e) CNC_{1300}, (f) CNT-CNC_{70}, (g) CNT-CNC_{800}, and (h) CNT-CNC_{1300}.

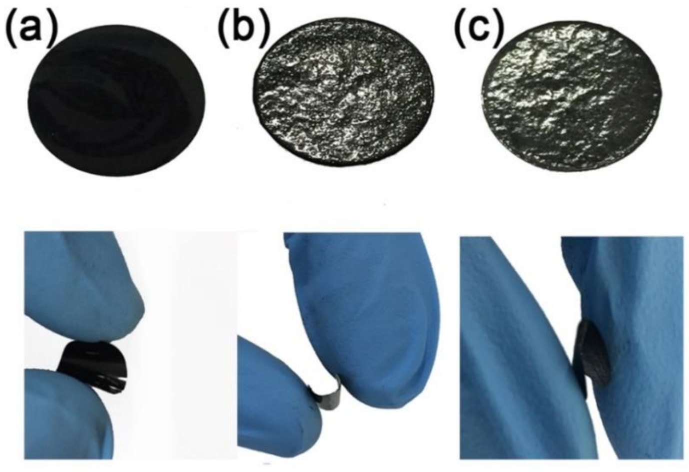

Figure. S5. Images of pliable electrodes: (a) CNT-CNC_{70}, (b) CNT-CNC_{800}, and (c) CNT-CNC_{1300}.

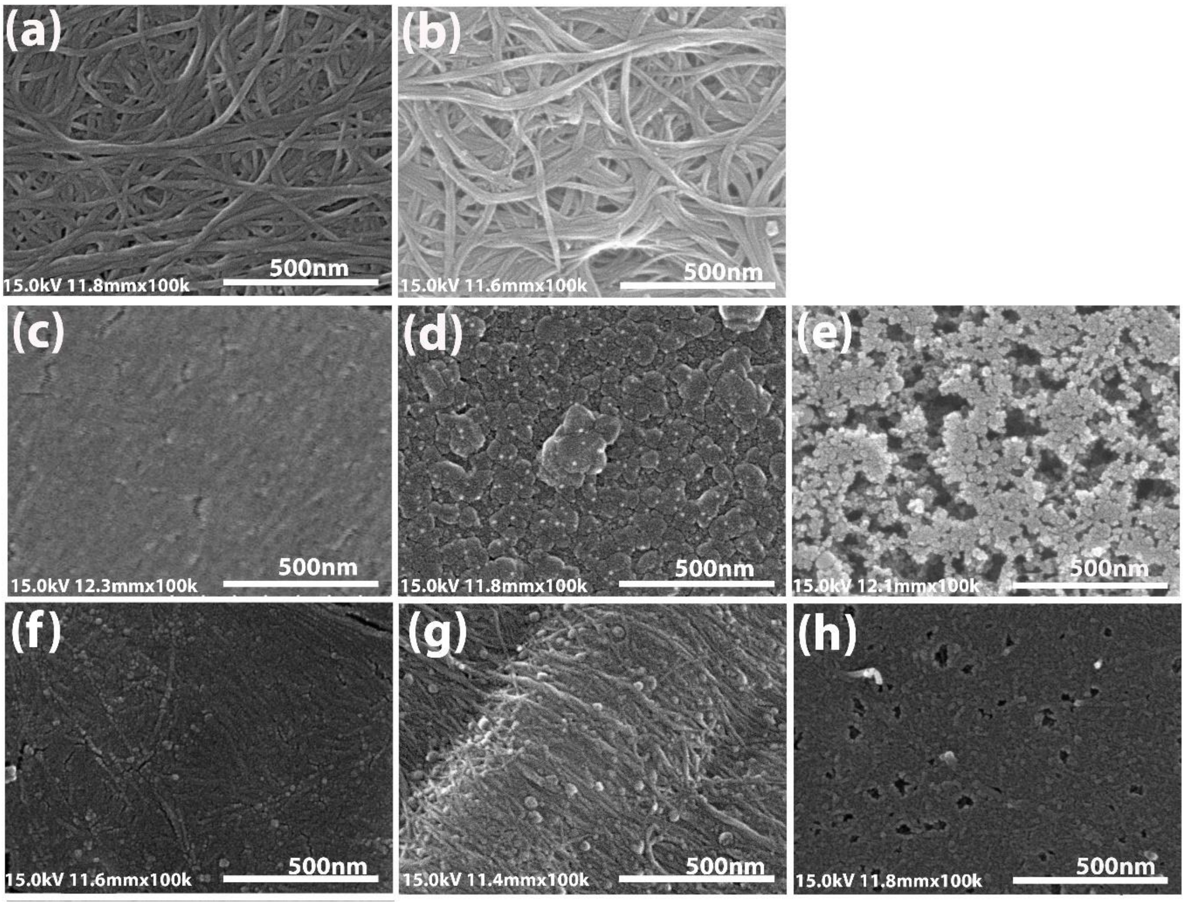

Figure. S6. SEM images (Surface section) of (a) CNT_{70}, (b) CNT_{800}, (c) CNC_{70}, (d) CNC_{800}, (e) CNC_{1300}, (f) CNT-CNC_{70}, (g) CNT-CNC_{800}, and (h) CNT-CNC_{1300}.

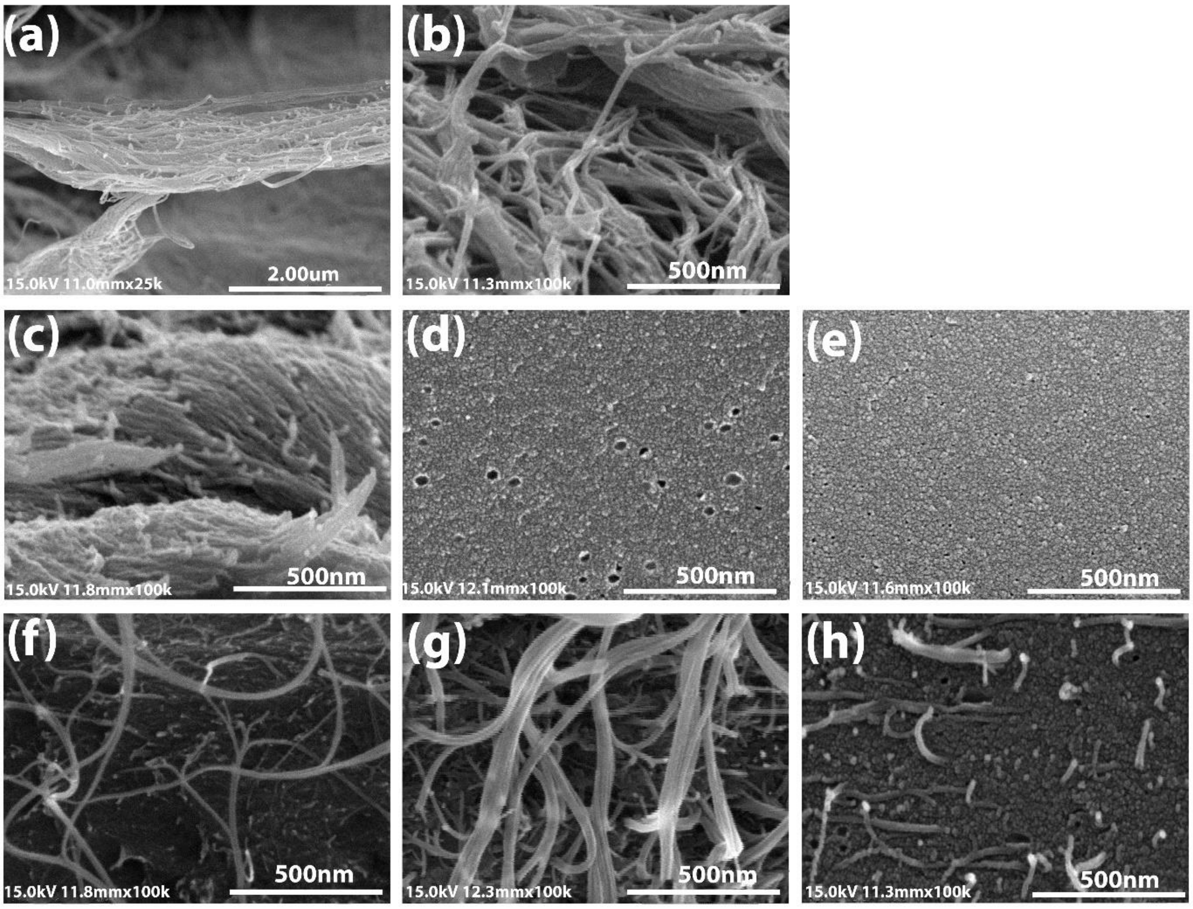

Figure. S7 . SEM images (Cross section) of (a) CNT_{70}, (b) CNT_{800}, (c) CNC_{70}, (d) CNC_{800}, (e) CNC_{1300}, (f) CNT-CNC_{70}, (g) CNT-CNC_{800}, and (h) CNT-CNC_{1300}.

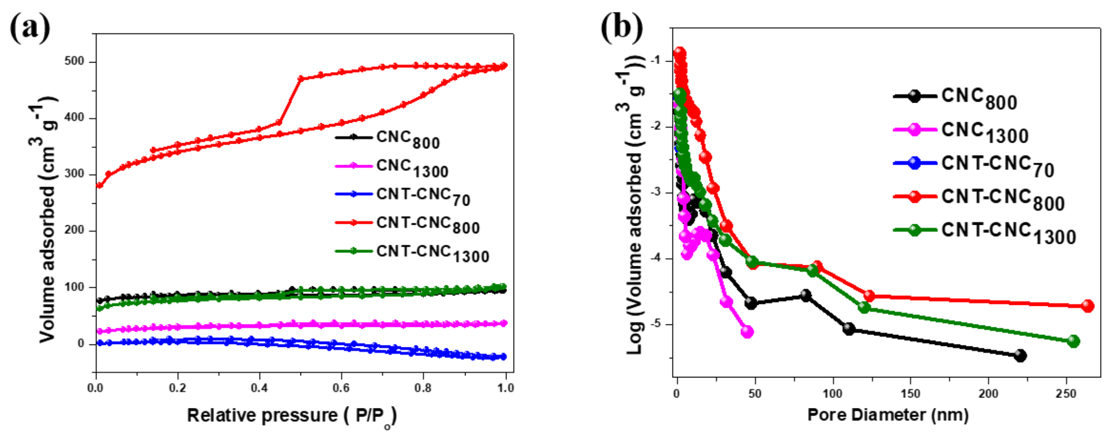

Figure. S8. BET results of CNC_{800}, CNC_{1300}, $CNT\text{-}CNC_{70}$, $CNC\text{-}CNT_{800}$, $CNC\text{-}CNT_{1300}$ using Barrett–Joyner–Halenda (BJH) model. (a) N_2-sorption isotherm curves and (b) Pore-size distribution curves.

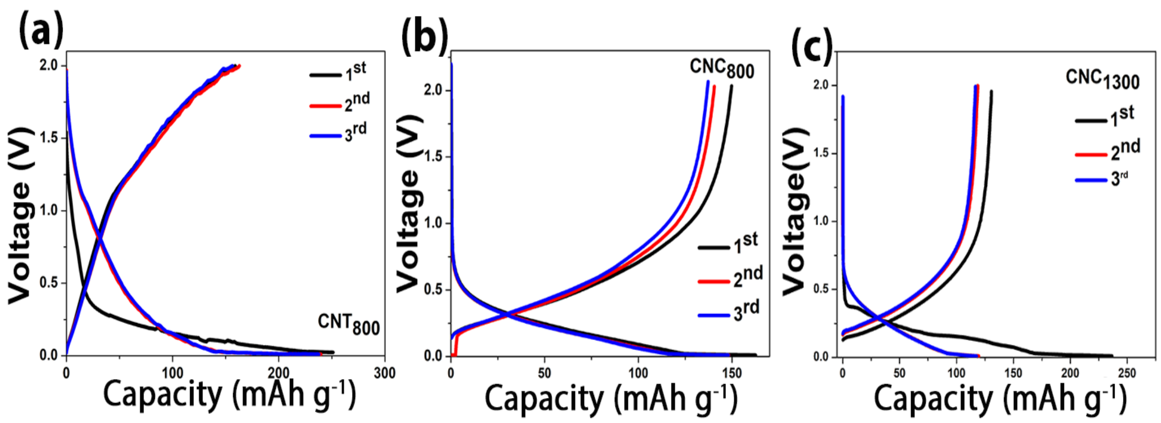

Figure. S9. Initial voltage profiles of (a) CNT_{800} (b) CNC_{800}, and (c) CNC_{1300}.

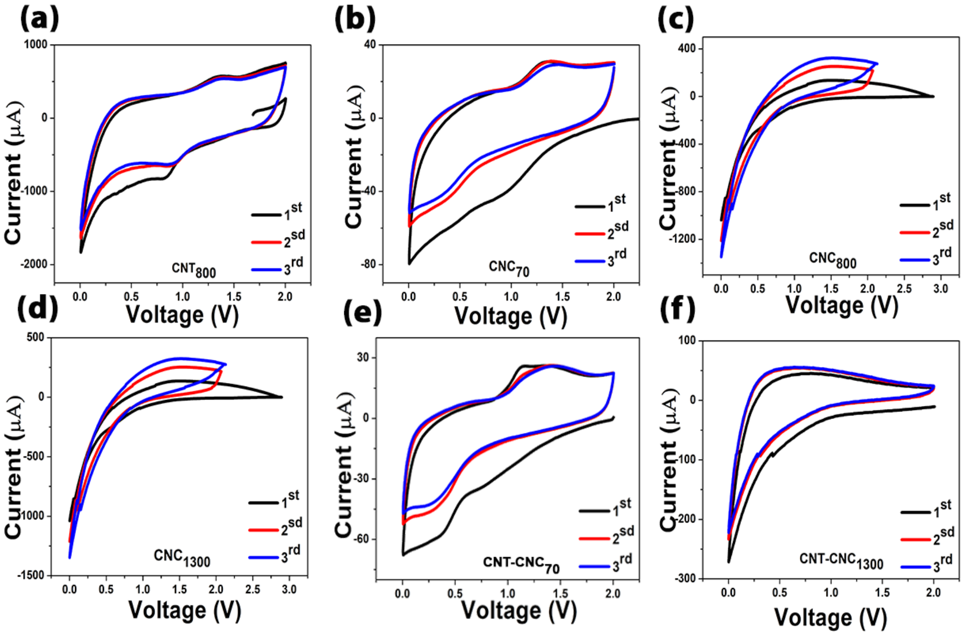

Figure. S10. Cyclic voltammograms of (a) CNT_{800}, (b) CNC_{70}, (c) CNC_{800}, (d) CNC_{1300}, (e)$CNT\text{-}CNC_{70}$, and (f) $CNT\text{-}CNC_{1300}$.

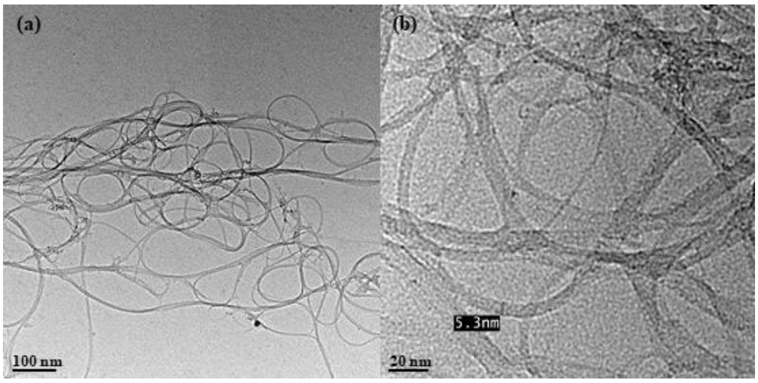

Figure. S11. TEM images of CNTs.

Table S4. Specific surface area of the as-prepared electrode films.

	BET Surface Area (m^2 g^{-1})	Pore Volume (cm^3 g^{-1})	Pore Size (nm)
CNC_{70}	-	-	-
CNC_{800}	285.80	0.1148	2.0501
CNC_{1300}	102.85	0.0562	2.1960
CNT-CNC_{70}	12.934	0.0044	2.1739
CNT-CNC_{800}	1184.2	0.7595	2.6257
CNT-CNC_{1300}	278.20	0.1201	2.2201

Table S5. Comparison of the performance for various flexible electrodes with CNTs.

Sample	Method	Current density	Capacity	Capacity retention after cycles	Ref.
Cu- nanowire/MWNT	Salt-assisted polyol method and dispersions	65 mA g^{-1}	370 mAh g^{-1} at 500th	90.2% (500th)	[7]
CNT-Film	Chemical vapor deposition and direct spinning	100 mA g^{-1}	446 mAh g^{-1} at 10th	48% (50th)	[8]
3D CNT/Cu mesh	Multi-stacking layers of free-standing CNTs	186 mA g^{-1}	312 mAh g^{-1} at 3rd	93% (50th)	[9]
ACNT/PEDOT	Chemical vapor-phase polymerization	0.1 mA cm^{-2}	265 mAh g^{-1} at 50th	90% (50th)	[10]
CNTs-GNS	Vacuum-assisted filtration	100 mA g^{-1}	330 mAh g^{-1} at 100th	92% (50th)	[11]
SWNT	Filtration method via positive pressure	0.08 mA cm^{-2}	173 mAh g^{-1} at 100th	86% (100th)	[12]
CNT/Carbon black/Triton X-100	Dispersion and Vacuum filtration	150 mA g^{-1}	270 mAh g^{-1} at 40th	58% (50th)	[13]
SWCNT/SnO_2	Polyol method and vacuum filtration	25 mA g^{-1}	454 mAh g^{-1} at 100th	86% (65th)	[14]
**CNT-CNCs**	**Dispersion and Free-standing**	232 mA g^{-1}	**450 mAh g^{-1} at 120th**	**89% (120th)**	**Our work**

References

1. Hamedi, M.M.; Hajian, A.; Fall, A.B.; Håkansson, K.; Salajkova, M.; Lundell, F.; Wågberg, L.; Berglund, L.A. Highly Conducting, Strong Nanocomposites Based on Nanocellulose-Assisted Aqueous Dispersions of Single-Wall Carbon Nanotubes. *ACS nano* **2014**, *8*, 2467-2476.
2. Cui, H.; Yan, X.; Monasterio, M.; Xing, F. Effects of Various Surfactants on the Dispersion of MWCNTs-OH in Aqueous Solution. *Nanomaterials* **2017**, *7*.
3. Sirviö, J.A.; Visanko, M.; Heiskanen, J.P.; Liimatainen, H. UV-absorbing cellulose nanocrystals as functional reinforcing fillers in polymer nanocomposite films. *J Mater Chem A* **2016**, *4*, 6368-6375.
4. Kumar, B.; Castro, M.; Feller, J.F. Controlled conductive junction gap for chitosan–carbon nanotube quantum resistive vapour sensors. *J Mater Chem* **2012**, *22*, 10656-10664.
5. Ramesh, S.; Ericson, L.M.; Davis, V.A.; Saini, R.K.; Kittrell, C.; Pasquali, M.; Billups, W.E.; Adams, W.W.; Hauge, R.H.; Smalley, R.E. Dissolution of Pristine Single Walled Carbon Nanotubes in Superacids by Direct Protonation. *J Phys Chem B* **2004**, *108*, 8794-8798.
6. Hajian, A.; Lindström, S.B.; Pettersson, T.; Hamedi, M.M.; Wågberg, L. Understanding the Dispersive Action of Nanocellulose for Carbon Nanomaterials. *Nano Lett* **2017**, *17*, 1439-1447.
7. Yin, Z.; Cho, S.; You, D.-J.; Ahn, Y.-k.; Yoo, J.; Kim, Y.S. Copper nanowire/multi-walled carbon nanotube composites as all-nanowire flexible electrode for fast-charging/discharging lithium-ion battery. *Nano Res* **2018**, *11*, 769-779.
8. Yoon, S.; Lee, S.; Kim, S.; Park, K.-W.; Cho, D.; Jeong, Y. Carbon nanotube film anodes for flexible lithium ion batteries. *J Power Sources* **2015**, *279*, 495-501.
9. Kang, C.; Patel, M.; Rangasamy, B.; Jung, K.-N.; Xia, C.; Shi, S.; Choi, W. Three-dimensional carbon nanotubes for high capacity lithium-ion batteries. *J Power Sources* **2015**, *299*, 465-471.
10. Chen, J.; Liu, Y.; Minett, A.I.; Lynam, C.; Wang, J.; Wallace, G.G. Flexible, Aligned Carbon Nanotube/Conducting Polymer Electrodes for a Lithium-Ion Battery. *Chem Mater* **2007**, *19*, 3595-3597.
11. Hu, Y.; Li, X.; Wang, J.; Li, R.; Sun, X. Free-standing graphene–carbon nanotube hybrid papers used as current collector and binder free anodes for lithium ion batteries. *J. Power Sources* **2013**, *237*, 41-46.
12. Ng, S.H.; Wang, J.; Guo, Z.P.; Chen, J.; Wang, G.X.; Liu, H.K. Single wall carbon nanotube paper as anode for lithium-ion battery. *Electrochim Acta* **2005**, *51*, 23-28.
13. Chew, S.Y.; Ng, S.H.; Wang, J.; Novák, P.; Krumeich, F.; Chou, S.L.; Chen, J.; Liu, H.K. Flexible free-standing carbon nanotube films for model lithium-ion batteries. *Carbon* **2009**, *47*, 2976-2983.
14. Noerochim, L.; Wang, J.-Z.; Chou, S.-L.; Wexler, D.; Liu, H.-K. Free-standing single-walled

carbon nanotube/SnO2 anode paper for flexible lithium-ion batteries. *Carbon* **2012**, *50*, 1289-1297.
